# Comparative validation of the knee inflammation MRI scoring system and the MRI osteoarthritis knee score for semi-quantitative assessment of bone marrow lesions and synovitis-effusion in osteoarthritis: an international multi-reader exercise

**DOI:** 10.1177/1759720X231171766

**Published:** 2023-07-12

**Authors:** Walter P. Maksymowych, Jacob L. Jaremko, Susanne J. Pedersen, Iris Eshed, Ulrich Weber, Andrew McReynolds, Paul Bird, Stephanie Wichuk, Robert G. Lambert

**Affiliations:** Department of Medicine, University of Alberta, 568 Heritage Medical Research Building, University of Alberta, Edmonton, AB T6R 2G8, Canada; CARE Arthritis, Edmonton, AB, Canada; Department of Radiology and Diagnostic Imaging, University of Alberta, Edmonton, AB, Canada; Medical Imaging Consultants, Edmonton, AB, Canada; Copenhagen Center for Arthritis Research, Center for Rheumatology and Spine Diseases, Rigshospitalet, Copenhagen, Denmark; Sheba Medical Center, Sackler School of Medicine, Tel-Aviv University, Tel Aviv, Israel; Practice Buchsbaum, Schaffhausen, Switzerland; Department of Radiology and Diagnostic Imaging, University of Alberta Hospital, Edmonton, AB, Canada; Division of Medicine, University of New South Wales, Sydney, NSW, Australia; Department of Medicine, University of Alberta, Edmonton, AB, Canada; Department of Radiology and Diagnostic Imaging, University of Alberta, Edmonton, AB, Canada; Medical Imaging Consultants, Edmonton, AB, Canada

**Keywords:** bone marrow lesion, effusion, magnetic resonance imaging, osteoarthritis, scoring system, synovitis, validation

## Abstract

**Background:**

Bone marrow lesions (BMLs) and synovitis on magnetic resonance imaging (MRI) are associated with symptoms and predict degeneration of articular cartilage in osteoarthritis (OA). Validated methods for their semiquantitative assessment on MRI are available, but they all have similar scoring designs and questionable sensitivity to change. New scoring methods with completely different designs need to be developed and compared to existing methods.

**Objectives:**

To compare the performance of new web-based versions of the Knee Inflammation MRI Scoring System (KIMRISS) with the MRI OA Knee Score (MOAKS) for quantification of BMLs and synovitis-effusion (S-E).

**Design:**

Retrospective follow-up cohort.

**Methods:**

We designed web-based overlays outlining regions in the knee that are scored for BML in MOAKS and KIMRISS. For KIMRISS, both BML and S-E are scored on consecutive sagittal slices. The performance of these methods was compared in an international reading exercise of 8 readers evaluating 60 pairs of scans conducted 1 year apart from cases recruited to the OA Initiative (OAI) cohort. Interobserver reliability for baseline status and baseline to 1 year change in BML and S-E was assessed by intra-class correlation coefficient (ICC) and smallest detectable change (SDC). Feasibility was assessed using the System Usability Scale (SUS).

**Results:**

Mean change in BML and S-E was minimal over 1 year. Pre-specified targets for acceptable reliability (ICC ⩾ 0.80 and ⩾ 0.70 for status and change scores, respectively) were achieved more frequently for KIMRISS for both BML and synovitis. Mean (95% CI) ICC for change in BML was 0.88 (0.83–0.92) and 0.69 (0.60–0.78) for KIMRISS and MOAKS, respectively. KIMRISS mean SUS usability score was 85.7 and at the 95th centile of ranking for usability versus a score of 55.4 and 20th centile for MOAKS.

**Conclusion:**

KIMRISS had superior performance metrics to MOAKS for quantification of BML and S-E. Both methods should be further compared in trials of new therapies for OA.

## Introduction

Bone marrow lesions (BMLs) and synovitis are frequently observed on magnetic resonance imaging (MRI) in osteoarthritis (OA). They associate strongly with knee pain structural degeneration of the articular cartilage and predict progression to joint replacement.^1–4^ In animal models of OA, BMLs precede cartilage degeneration, thus providing an early indication of OA.^5,6^ In humans, when found in early OA, they predict regional cartilage loss,^7^ but additional data indicate that BML are found predominantly at sites of cartilage loss suggesting that they represent a bone response to abnormal loading.^8^ Synovial activation in OA is thought to be a secondary phenomenon related to cartilage deterioration, but there is also evidence that synovitis plays a role in the progression of cartilage loss in knee OA.^9^ Several molecules found in inflamed synovium of OA patients have been investigated in recent years and therapeutically targeted primarily for the inflammatory manifestations of OA such as interleukin (IL)-1, tumor necrosis factor (TNF)-, and iNOS inhibitors.^10,11^

Because these MRI features are independently associated with the severity and progression of OA,^12^ they constitute relevant targets for therapeutic intervention. Moreover, they constitute objective outcome measures in randomized controlled trials of OA which may be complementary to the patient self-reported clinical outcomes assessing domains such as pain and functional impairment commonly used in OA trials. However, current clinical trials continue to focus predominantly on patient self-reported measures despite documentation of potential confounders, such as pain hypersensitization, and high placebo response rates. Few trials have targeted reducing the size of BML and degree of synovitis on MRI for the treatment of OA,^13–16^ possibly because existing MRI-based scoring methods have not been specifically designed for the purpose of demonstrating sensitivity to change for these lesions. In a recent landmark review, it was stated that ‘changes in approaches to evaluating efficacy will increase the chances of demonstrating efficacy of promising treatments for OA.^17^’ This review highlighted the limitations inherent to the use of self-reported symptoms, especially pain, in trials of OA and the possibility that improvement in OA symptoms may be accompanied not by a reduction in pain but rather by an improvement in the patient’s ability to do particular activities, leading to increased activity levels. These authors suggested alternative approaches such as focusing on innervated structures in the OA joint including bone and synovium, in which pathology includes BML and synovitis, respectively, since studies have shown that both predict subsequent cartilage loss or structural deterioration.^18,19^

A variety of semiquantitative knee OA scoring systems have been developed to assess BML and synovitis, the most commonly used including the Whole-Organ MRI Score (WORMS), Boston-Leeds Osteoarthritis Knee Score (BLOKS), and MRI Osteoarthritis Knee Score (MOAKS).^20–23^ Synovitis-effusion (S-E) has been assessed qualitatively according to a 0–3 grade on axial images that is based on the degree of distension of the medial and lateral patellar recesses. S-E in other locations, such as the suprapatellar bursa, are not fully assessed. The design of this scoring method for S-E is ill-suited for detecting change since assessment is a qualitative assessment of only three grades limited to two synovial recesses of the knee on a single orientation. Each method assesses BML by dividing the knee into subregions and then grading size of BML according to a 0–3 grading scale based on the percentage volume of a region filled by a BML. It has been recognized that substantive changes in the extent of BML may be evident without this leading to a change in grade of lesion due to the relatively large volume of certain subregions, especially those in the femoral and tibial condyles.^23^ This is problematic because BMLs are typically observed in the subchondral regions of the condyles. Feasibility of the MOAKS method may also be questioned because the boundary lines that define the subregions have to be positioned on 3 orientations of the MRI scan of the knee joint, guidelines for placement of boundary lines are lacking for anatomical variation and non-orthogonal images, and knowledge transfer tools and web-based methods for direct data entry are lacking.

We have developed a novel scoring methodology to assess BML and S-E, the Knee Inflammation MRI Scoring System (KIMRISS), which employs interactive web-based image overlays for each articular surface in the knee on a fluid sensitive MRI sequence in the sagittal orientation.^24^ The primary aims of this new semi-quantitative method were to enhance feasibility of scoring and sensitivity to change. The first version of the overlays divided subarticular bone into 763 ∼1 × 1 cm regions in the femur, tibia, and patella, each region being scored either 0, by default, or 1 if there is BML after the reader touches or mouse-clicks the BML-containing region which causes it to change color onscreen for feedback. We demonstrated that KIMRISS was more reliable and responsive than MOAKS for detection of change in BML in Osteoarthritis Initiative observational data over a 1-year time frame and in a 12-week open label trial of adalimumab for inflammatory OA of the knee.^24^ We also validated a real-time iterative calibration (RETIC) online tool for KIMRISS where readers can improve their scoring proficiency prior to formal scoring exercises.^25,26^

We identified several limitations of version 1 of these overlays, primarily limitation of feasibility in using the overlays. Positioning of the overlays lacked precision for the different contours of the articulating bones and required frequent repositioning. The overlays included non-articular regions in the femoral condyles and tibial plateau raising concerns that, despite instructions to the contrary, observers might erroneously score regions that are not relevant to knee OA. Methodology for scoring also had to correct for inter-individual differences in the size of the knee. Consequently, the software used to place the overlays, along with guidelines for how to use the system, were redesigned to address these concerns and a new RETIC module was created. We also revised the scoring of S-E so that assessment was conducted in four pre-defined areas on *consecutive* sagittal slices in order to enhance sensitivity to change. We also designed a new web-based overlay to facilitate delineation of the boundaries of the subregions in MOAKS to enhance the scoring of BML and thereby optimize the comparative validation between the two methods. We aimed to validate this revised version of KIMRISS by comparing it with the enhanced version of MOAKS for inter-reader agreement for status and change scores, sensitivity to change, and feasibility.

## Materials and methods

### MRI scoring methodologies for BMLs and synovitis-effusion: KIMRISS

**Figure 1.**
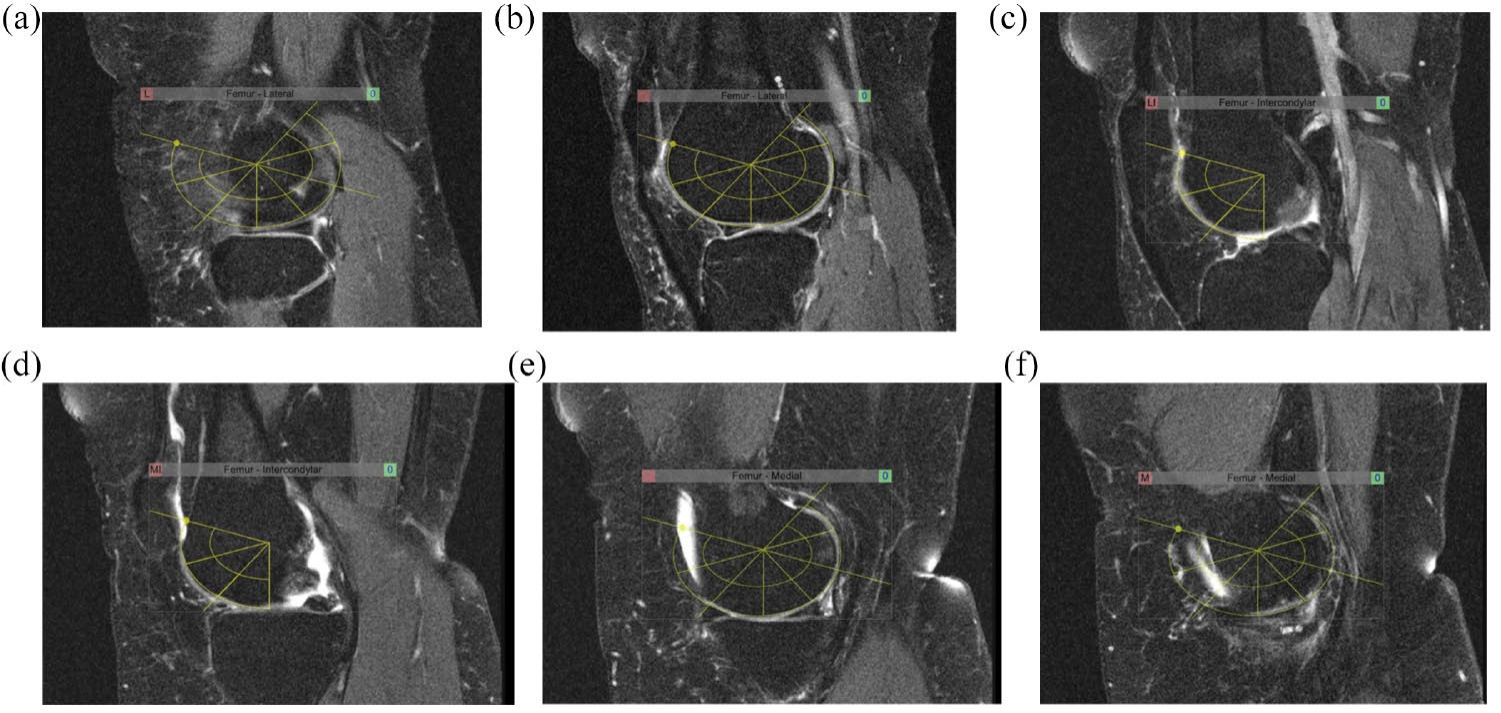
Femoral condyle anchors and overlays for the scoring of BML in the femoral condyles. *Femoral condyle anchors* are set in a stepwise manner while scrolling across images in the sagittal orientation from the lateral to the medial femoral condyles according to the following steps: (1) Set the lateral edge of the lateral condyle anchor on the most lateral sagittal slice where a portion of the articular surface of the lateral condyle is visible (Figure 1(a). Lateral edge lateral femoral condyle anchor). (2) Set the positional anchor of the lateral condyle on the sagittal slice where the lateral condyle appears largest. This anchor may be re-sized and re-positioned to match the articular surface (Figure 1(b) Lateral femoral condyle positional anchor). (3) Set the lateral intercondylar anchor on the sagittal slice where the posterior cruciate ligament (PCL) first becomes visible when scrolling from the lateral to the medial portion of the intercondylar region (Figure 1(c) Lateral intercondylar anchor). This anchor should not be re-sized but can be repositioned to match the articular surface. (4). Set the medial intercondylar anchor on the last sagittal slice where the PCL is still visible when scrolling from the lateral to the medial portion of the intercondylar region (Figure 1(d) Medial intercondylar anchor). This anchor should not be re-sized but can be repositioned to match the articular surface. (5) Set the positional anchor of the medial condyle on the sagittal slice where the medial condyle appears largest (Figure 1(e) Medial femoral condyle positional anchor). This anchor should not be re-sized but can be repositioned to match the articular surface. (6) Set the medial edge of the medial condyle anchor on the most medial sagittal slice where a portion of the articular surface of the medial condyle is still visible (Figure 1(f) Medial edge medial femoral condyle anchor). This anchor should not be re-sized but can be repositioned to match the articular surface.

The KIMRISS method scores BML in the femoral condyles, tibial plateau, and patella as well as S-E on sagittal MR images of fluid sensitive sequences. Subchondral cysts are not scored. A BML is defined as an area of altered signal compared to adjacent normal subchondral marrow that has hypointense or intermediate signal on T1-weighted imaging (T1w) and a hyperintense signal on fluid sensitive sequences, such as fat suppressed (FS) T2-weighted imaging (T2w) or short-tau inversion recovery (STIR). The femoral condyles, tibial plateaus and patella are divided into sectors and a template outlining these sectors, comprised of a web-based electronic overlay, is used for ease of scoring. The presence of BML in a sector is recorded in a binary manner as present or absent. The overlays follow the contour of subchondral bone and are set to record lesions from lateral to medial edges of the respective bones on consecutive sagittal slices after setting lateral and medial edge anchors. Overlay anchors are also set for the largest size of the respective bone (positional anchors). The overlays are positioned and may be re-sized to match the articular surface of the respective bone. The methodology is outlined in a series of steps in Figure 1.

Because the size of the knee varies from person to person, and the number of sagittal slices and/or slice thickness may also be variable, a reference number of sagittal slices is evaluated, and scores are automatically prorated if there are a different number of sagittal slices than those allocated for each region of the femur. These reference numbers are as follows: 10 sagittal slices for the lateral femoral condyle, 8 sagittal slices for the medial femoral condyle, 4 sagittal slices for the intercondylar femoral region. The femoral condylar overlays each comprise 14 small sectors of comparable volume per sagittal slice, while the intercondylar overlay comprises 6 sectors per sagittal slice. BML are scored directly online on a web-based interface as present ‘1’ or absent ‘0’ in each sector. This framework for scoring femoral BML leads to the following scoring ranges: Lateral Condyle (0–140), Medial Condyle (0–112), Inter-condylar Femur (0–24), and Total Femoral (0–276).

**Figure 2.**
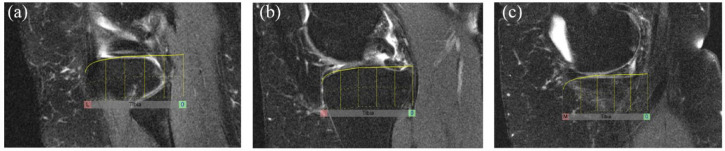
Tibial plateau anchors and overlays for the scoring of BML in the tibial plateau. *Tibial Plateau Anchors* are set in a stepwise manner while scrolling across images in the sagittal orientation from the lateral to the medial edges according to the following steps: 1. Set the lateral edge of the tibial plateau anchor on the most lateral sagittal slice where a portion of the surface of the tibial plateau is still visible (Figure 2(a). Lateral edge of Tibial Plateau anchor). The anterior curve of the overlay should match the anterior surface of the tibial plateau. 2. Set the positional anchor on the sagittal slice that includes the maximal antero-posterior length of the tibial plateau (Figure 2(b). Tibial Plateau Positional Anchor). The anterior curve of the overlay should match the anterior surface of the tibial plateau. The overlay may be re-positioned but not resized. 3. Set the medial edge of the tibial plateau anchor on the most medial sagittal slice where a portion of the surface of the tibial plateau is still visible (Figure 2(c). Medial Edge of Tibial Plateau anchor). The overlay may be re-positioned but not resized.

*Tibial plateau anchors* are set in a stepwise manner while scrolling across images in the sagittal orientation from the lateral to the medial edges of the tibial plateau according to the steps outlined in Figure 2. The reference number of sagittal slices that are scored for the tibial plateau is 20 and the tibial overlay comprises 10 sectors per sagittal slice leading to a scoring range for the tibial plateau of 0–200. Scores are automatically prorated if there are a different number of sagittal slices than those allocated for the tibial plateau.

**Figure 3.**
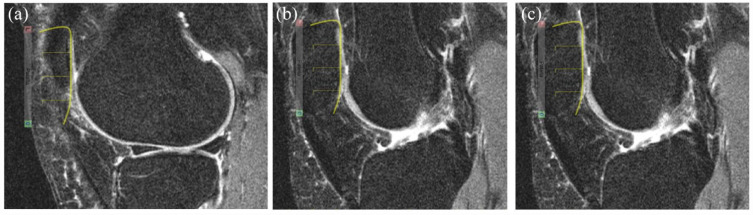
Patellar anchors and overlays for the scoring of BML in the patella. *Patellar Anchors* are set in a stepwise manner while scrolling across images in the sagittal orientation from the lateral to the medial edges according to the following steps: 1. Set the lateral edge of the patellar anchor on the most lateral sagittal slice where a portion of the patellar articular surface is still visible (Figure 3(a). Lateral edge of Patellar anchor). The superior curve of the overlay should match the superior surface of the patella. 2. Set the positional anchor on the sagittal slice that includes the largest size of the patella (Figure 3(b). Patellar Positional Anchor). The superior curve of the overlay should match the superior surface of the patella. The overlay may be re-positioned but not resized. 3. Set the medial edge of the patellar anchor on the most medial sagittal slice where a portion of the patellar articular surface is still visible (Figure 3(c). Medial Edge of Patella anchor). The overlay may be re-positioned but not resized.

*Patellar anchors* are set in a stepwise manner while scrolling in the sagittal orientation from the lateral to the medial edges of the patella according to the steps outlined in Figure 3. The reference number of sagittal slices that are scored for the patella is six and the patellar overlay comprises four sectors per sagittal slice leading to a **scoring range for the patella of 0-24**. Scores are automatically prorated if there are a different number of sagittal slices than those allocated for the patella.

#### The total scoring range for BML in the entire knee joint is 0–500

Knee Synovitis-Effusion (S-E) is evaluated on every sagittal slice where S-E is present. It is assessed on fluid sensitive sequences without contrast enhancement, and it is often not possible to distinguish synovitis from effusion. S-E increase the depth of synovial recesses. Therefore, to characterize S-E, the depth (short axis), and not the length (long axis), of each recess is measured by using a draw ruler available in all standard medical imaging viewer software. Measurements of depth are assigned the following scores:

i. Score 0 = 0 mm – 1.9 mm (normal)
ii. Score 1 = 2 mm – 4.9 mm
iii. Score 2 = 5 mm – 9.9 mm
iv. Score 3 = 10 mm – 19. 9 mm
v. Score 4 = ⩾ 20 mm

**Figure 4.**
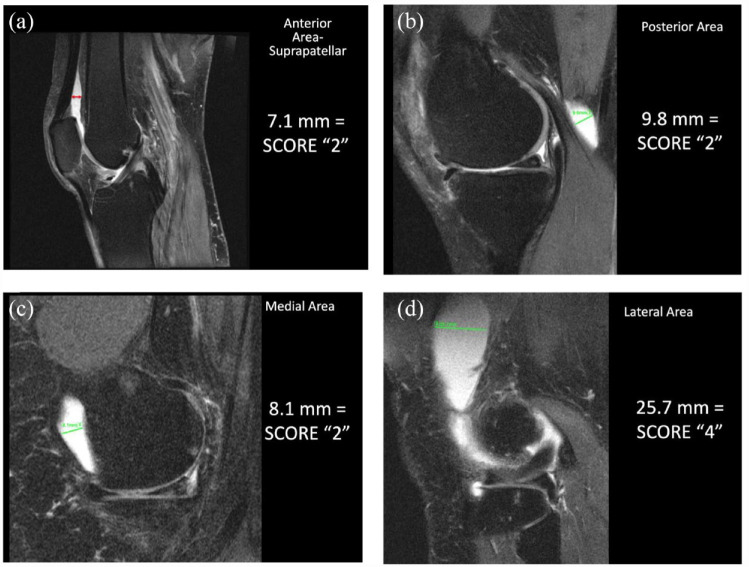
Assessment of synovitis-effusion by measuring the the depth (short axis) of synovial recesses in the anterior, posterior, medial, and lateral areas according to the following scoring scheme: score 0 = 0 mm – 1.9 mm (normal), score 1 = 2 mm – 4.9 mm, score 2 = 5 mm – 9.9 mm, score 3 = 10 mm – 19.9 mm, score 4 = ⩾ 20 mm. (a) Anterior area: Areas superior, inferior or posterior to the patella (patella visible) (includes supra-, retro-, infra-patellar areas (including Hoffa’s synovitis) and anterior to the mid-coronal plane of the tibial plateau (including intercondylar synovitis). (b) Posterior area: Areas posterior to the mid-coronal plane of the tibial plateau (including intercondylar synovitis and Baker’s cyst). (c) Medial area: Areas medial to the patella (patella not visible) and anterior to the mid-coronal plane of the tibial plateau. (d) Lateral area: Areas lateral to the patella (patella not visible) and anterior to mid-coronal plane of the tibial plateau.

For each sagittal slice, four compartments are assessed and the compartment with the greatest depth of S-E is determined and scored as outlined in Figure 4. The reference number of sagittal slices that are scored for S-E is 25 and since the scoring range for depth of S-E is 0–4, this leads to a **total scoring range for the entire knee joint of 0-100**. Scores are automatically prorated if there are a different number of sagittal slices.

##### Knowledge transfer and calibration tools

**Figure 5.**
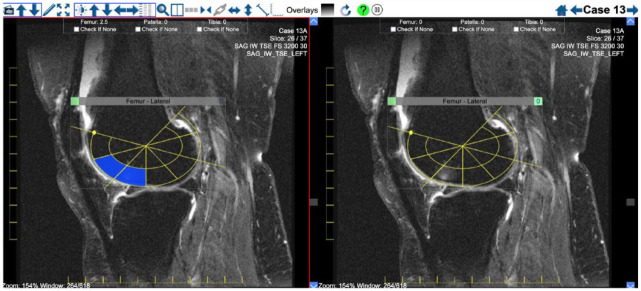
The KMIRSS Real-Time Iterative Calibration (RETIC) module for calibration of readers scoring BML using the KIMRISS method (www.carearthritis.com/mriportal/kimriss). This is comprised of 20 cases with knee OA, each with baseline and 1-year scans scored by the 3 developers of KIMRISS. BML observed in these cases are scored blinded to time point directly on a web-based interface as present ‘1’ or absent ‘0’ in each sector. Discordance or concordance for presence/absence of BML with developer consensus assignment at the level of individual sectors is indicated by the color change as the reader mouse-clicks each individual subregion within the overlay as the reader scores the case. Reliability for baseline, 1 year, and baseline to 1-year change in BML score compared to developer scores is provided online in real time after 10 and then again after 20 cases have been scored according to the intra-class correlation coefficient (ICC). This allows progressive learning and calibration for setting the threshold of sensitivity for abnormal MRI signal with each new case in the RETIC module. In this example, there is BML in the lateral femoral condyle (right panel) which has been recorded concordantly (blue color coding in left panel) with KIMRISS developer scoring. A red color would indicate discordant scoring.

A powerpoint module and YouTube video have been developed illustrating the KIMRISS method and the approach to the setting of anchors that can be accessed online.^26^ A real-time iterative calibration module (RETIC) for calibration of readers intending to use KIMRISS has also been developed and is available online at the same website (Figure 5). Readers have to achieve scoring proficiency targets according to the intra-class correlation coefficient (ICC; ⩾0.80 for status score, ⩾0.70 for change score, for both BML and synovitis-effusion) that are comparable to those achieved by the developers (0.90 and 0.88 for status and change scores, respectively) before embarking on any formal scoring exercise. The lower target ICC for change versus status score reflects the small degree of change in BML observed in patients with OA, even over time frames as long as 1 year, and the lack of a treatment with major impact on BML.

### MRI scoring methodologies for BMLs and synovitis-effusion: MOAKS

The MOAKS method has been described in detail in previous reports and is summarized in the supplemental section.^22^

#### Knowledge transfer and calibration tools

**Figure 6.**
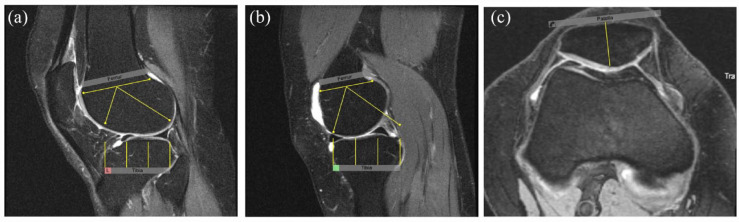
The web-based overlays for delineating the boundaries of subregions of the knee that are scored for BML using the MOAKS method. (a) Lateral anchor, (b) Medial Anchor and (c) Patellar anchor.

Knowledge transfer tools detailing the methodology for scoring MOAKS are comprised of two manuscripts (personal communication from MOAKS developer, Dr. Frank Roemer).^22,27^ Consequently, we designed a new powerpoint module based on these manuscripts as well as new web-based overlays and a new scoring interface to enable direct online data entry for recording BML in the different anatomical regions stipulated in MOAKS as illustrated further online^28^ (Figure 6).

##### MRI scans available

We used publicly available data (release 18) from the Osteoarthritis Initiative (OAI), a multicentre prospective observational study of 4796 patients with, or at risk for, OA and currently in its 14th year of follow-up.^29^ The OAI study recruited 1396 participants with symptomatic knee OA (frequent pain and definite radiographic signs) of at least one knee (‘progression cohort’) and 3278 with an increased risk of developing symptomatic OA. Patients received standard of care treatment for OA that included acetaminophen, non-steroidal anti-inflammatory agents, and intra-articular steroid. MRI scans from a subset of these cases have already been scored using the MOAKS method by an OAI central reader. Our goal was to compare the feasibility, reliability, and sensitivity to change of KIMRISS versus MOAKS in knees that demonstrated evidence of BML and S-E at baseline but were not considered to reflect end-stage disease at the cusp of requiring arthroplasty. Consequently, we selected the first 60 cases that met the following criteria: (1) Kellgren-Lawrence grade ⩽ 3 on radiographs, (2) available MRI scans from both time points of any 1-year interval and scored centrally according to the MOAKS method, and (3) MOAKS BML score ⩾ 1 and MOAKS S-E score ⩾ 1 at start of or at the 1-year follow-up according to central read.

##### Reading exercises

The 8 MRI readers included the 3 developers of the KIMRISS method (WPM (rheumatologist), RGL (MSK radiologist), JJ (MSK radiologist)), 3 experienced readers, comprised of 2 rheumatologists and one musculoskeletal radiologist, with > 10-years of experience in development and validation of MRI-based scoring instruments that included prior reading exercises evaluating KIMRISS and MOAKS, and 2 inexperienced readers comprised of 1 rheumatologist and 1 radiology fellow with no prior experience with the use of either the KIMRISS or MOAKS methods. Prior to the assessment of the 60 cases from the OAI dataset, all readers reviewed the manuscripts describing the scoring methods and the powerpoint modules summarizing the scoring methods with examples of images scored by consensus reads, and then scored cases in the RETIC module aimed at achieving target ICC of ⩾0.80 for status and ⩾0.70 for change scores in BML. The 60 cases were then read blinded to time point. Two different reading IDs were assigned per case to denote scoring with either the KIMRISS or MOAKS method and the order of reads was randomized. Pre-specified acceptable targets for reader reliability were the same as those specified for the RETIC module, namely, an ICC of ⩾0.80 for baseline status scores and ⩾0.70 for change scores for each of BML and synovitis-effusion.

##### Assessment of feasibility

Feasibility of KIMRISS and MOAKS was assessed by recording the time expended on the reading of each case, which was done automatically by the reading software. Readers also completed the System Usability Scale (SUS)^30^ at the completion of the exercise (www.usability.gov). The SUS is a simple, 10-item attitude Likert-type scale giving a global view of subjective assessments of usability. It has been widely used in the evaluation of a range of systems and this has led to normative data that allow SUS ratings to be positioned relative to other systems.^31^ Because it yields a single score on a scale of 0–100, with higher scores indicating higher perceived usability, it can be used to even compare systems that are outwardly dissimilar. Its psychometric properties have been extensively studied.^32–35^ Normative data are available based on scores from 11,855 individual SUS assessments from 166 industrial usability studies.^36^ Raw SUS scores can be converted into percentile ranks.^37^ The 50th percentile score is 68 and is generally regarded as the cut-off for an instrument likely to be widely applied.

##### Association of BML and synovitis-effusion to outcomes

Although this work focused primarily on feasibility, reliability, and sensitivity to change, as a secondary analysis we compared the construct validity of the two scoring methods by analyzing Spearman’s correlations between BML or S-E scores and the Western Ontario and McMaster Universities (WOMAC) pain score developed for patients with knee or hip OA, which has a scoring range of 0–20.^38^

##### Statistics

Descriptive statistics were reported as mean ± SD. Given the large scoring ranges of both MOAKS and KIMRISS BML scores, we treated each as a quasi-continuous variable for analysis, and for simplicity, considered the whole-joint total BML score for most analyses. For assessment of interobserver reliability, we used the single measure intraclass correlation coefficient (ICC), absolute agreement definition.^39^ We also computed smallest detectable change (SDC) based on the 95% confidence interval (CI) of interobserver variability of change scores.^40^ For responsiveness, we computed standardized response means (SRM) and performed paired Student’s t-tests to assess for statistical significance of observed changes in BML and synovitis-effusion by KIMRISS or MOAKS. We explored associations between 1-year change in BML or S-E and corresponding 1-year change in WOMAC pain using Spearman’s correlation and, also, using multivariate regression that included age, gender, body mass index (BMI), and Kellgren-Lawrence radiographic grade as covariates. For assessment of feasibility, we calculated individual reader SUS scores for the KIMRISS, the MOAKS as currently available without the web-based interface that we developed, and the MOAKS with the web-based reading interface. Raw scores were converted to percentile rankings.

## Results

### Patient characteristics and MRI scores

The baseline characteristics of cases whose scans were evaluated in this exercise were as follows: mean (SD) age of 61.9 (8.8) years, 16 (26.7%) males, WOMAC pain (range 0-20) (mean (SD)) 12.3 (11.9), and Kellgren-Lawrence grades 0 (10%), 1 (36.7%), 2 (53.3%) (supplementary Table 1). A small, though non-significant, reduction in mean BML score was noted at 1 year with both KIMRISS and MOAKS and the SDC was 1.1 for the MOAKS, which is 2.4% of the total scoring range, and 4.3 for KIMRISS, which is 0.9% of the total scoring range (Table 1). There was no change in MOAKS synovitis-effusion score and a slight increase in KIMRISS synovitis-effusion mean score of 1.1. Smallest detectable change for MOAKS synovitis-effusion score was 0.4 (13.3% of total scoring range) and 2.2 (2.2% of total scoring range) for KIMRISS synovitis-effusion score.

**Table 1.** MRI bone marrow lesion and synovitis-effusion scores at baseline and 1 year in cases selected from the Osteoarthritis Initiative and assessed using the KIMRISS and MOAKS MRI scoring methods.

Method	MRI feature	MRI Scores, mean (SD)			Range of Change Scores	SDC (% of maximum)	*p* value	SRM
		Baseline	1-year	Change				
MOAKS	BML^a^	4.2 (2.6)	3.7 (2.4)	–0.5 (2.1)	–10.0 to 2.2	1.1 (2.4%)	0.22	–0.24
	BML-WG^a^	3.9 (2.5)	3.4 (2.3)	–0.5 (2.1)	–9.9 to 2.0	1.1	0.15	–0.24
	Synovitis-effusion^b^	1.2 (0.7)	1.3 (0.8)	0.0 (0.5)	–1.5 to 1.3	0.4 (13.3%)	0.53	0.0
KIMRISS	BML^a^	22.1 (20.8)	19.1 (17.1)	–3.1 (14.6)	–68.3 to 16.2	4.3 (0.9%)	0.64	–0.21
	Synovitis-effusion^b^	21.8 (9.3)	22.9 (10.8)	1.1 (7.1)	–14.0 to 20.0	2.2 (2.2%)	0.46	0.15

BML, bone marrow lesion; SDC, smallest detectable change; SRM, standardized response mean; WG, within-grade scoring of BML.

a BML data from 5 expert readers.

b Synovitis-effusion data from 6 expert readers.

### Reliability for BML and synovitis-effusion MRI KIMRISS and MOAKS scores

Acceptable reliability for BML baseline status score (ICC > 0.80) using KIMRISS was achieved for all pair-wise comparisons of experienced readers (14/14) and for 1 of 13 pair-wise comparisons that included at least one inexperienced reader (Table 2). For MOAKS, this target was attained by only a single reader pair that comprised 2 experienced readers (Table 2 and supplementary Table 2). Spearman’s Rank correlations between KIMRISS and MOAKS status scores varied from 0.39 to 0.78 among all the reader pairs and all were highly significant (*p* < 0.0001) (supplementary Table 3). Acceptable reliability for change from baseline to 1-year score of BML (ICC > 0.70) was achieved for all 14 pair-wise comparisons of experienced readers using KIMRISS and, also, for 2 of the 13 pair-wise comparisons that included an inexperienced reader (Table 3). For MOAKS BML change score, acceptable reliability was achieved for 9/14 pair-wise comparisons between experienced readers and for 1 of the 13 pair-wise comparisons that included an inexperienced reader (Table 3 and supplementary Table 4). Spearman’s Rank correlations between KIMRISS and MOAKS change scores varied from 0.18 to 0.59 among all the reader pairs and most were highly significant (*p* < 0.0001) (supplementary Table 5).

**Table 2.** Inter-reader reliability for status (baseline scan) score in BML using the KIMRISS (A) and MOAKS (B) MRI scoring platforms in 60 cases selected from the Osteoarthritis Initiative database. Values in the table reflect pair-wise intraclass correlation coefficients (95% confidence intervals) and bolded values are those which attain the pre-specified target for acceptable reliability of > 0.80. Readers 1 and 8 are inexperienced readers.

	Reader 1	Reader 2	Reader 3	Reader 4	Reader 5	Reader 6	Reader 7
A. KIMRISS							
Reader 2	0.64 (0.12–0.83)						
Reader 3	**0.75 (0.28–0.89)**	**0.90 (0.83–0.94)**					
Reader 4	0.50 (0.04–0.74)	**0.93 (0.87–0.96)**	**0.81 (0.64–0.89)**				
Reader 5	0.57 (0.06–0.79)	**0.95 (0.92–0.97)**	**0.88 (0.75–0.94)**	**0.94 (0.90–0.96)**			
Reader 6	0.56 (0.07–0.79)	**0.93 (0.89–0.96)**	**0.86 (0.74–0.92)**	**0.93 (0.89–0.96)**	**0.94 (0.90–0.96)**		
Reader 7	0.60 (0.11–0.81)	**0.97 (0.95–0.98)**	**0.90 (0.82–0.94)**	**0.94 (0.90–0.97)**	**0.95 (0.92–0.97)**	**0.92 (0.87–0.95)**	
Reader 8	0.32 (-0.06–0.59)	0.61 (0.23–0.79)	0.54 (0.13–0.76)	**0.71 (0.34–086)**	0.65 (0.31–0.82)	0.63 (0.30–0.80)	**0.92 (0.87–0.95)**
**B. MOAKS.**							
Reader 2	0.53 (0.18–0.73)						
Reader 3	0.61 (0.28–0.78)	0.58 (0.38–0.73)					
Reader 4	0.76 (0.61–0.85)	0.71(0.43–0.84)	0.72 (0.52–0.84)				
Reader 5	0.55 (0.14v0.76)	0.78(0.65–0.86)	0.71 (0.55–0.81)	0.73 (0.34–0.87)			
Reader 6	0.63 (0.46–0.76)	0.61 (0.29–0.79)	0.66 (0.40–0.81)	0.75 (0.61–0.84)	0.58 (0.23–0.76)		
Reader 7	0.51 (0.10–0.74)	**0.82 (0.71–0.89)**	0.57 (0.37–0.72)	0.69 (0.26–0.86)	0.73 (0.58–0.83)	0.50 (0.18–0.76)	
Reader 8	0.28 (–0.05–0.54)	0.55 (0.16–0.76)	0.55 (0.21–0.74)	0.47 (–0.06–0.74)	0.67 (0.24–0.84)	0.43 (–0.06–0.72)	0.56 (0.31–0.73)

**Table 3.** Inter-reader reliability for baseline to 1-year change score in BML using the KIMRISS (A) and MOAKS (B) MRI scoring platforms in 60 cases selected from the Osteoarthritis Initiative database. Values in the table reflect pair-wise intraclass correlation coefficients (95% confidence intervals) and bolded values are those which attain the pre-specified target for acceptable reliability of > 0.70. Readers 1 and 8 are inexperienced readers.

	Reader 1	Reader 2	Reader 3	Reader 4	Reader 5	Reader 6	Reader 7
**A. KIMRISS.**							
Reader 2	**0.74 (0.60–0.83)**						
Reader 3	**0.81 (0.70–0.88)**	**0.90 (0.84–0.94)**					
Reader 4	0.61 (0.42–0.75)	**0.88 (0.81–0.93)**	**0.85 (0.76–0.91)**				
Reader 5	0.69 (0.53–0.80)	**0.92 (0.88–0.95)**	**0.80 (0.79–0.92)**	**0.90 (0.84–0.94)**			
Reader 6	**0.73 (0.59–0.83)**	**0.90 (0.84–0.94)**	**0.83 (073–0.89)**	**0.81 (0.70–0.88)**	**0.87 (0.79–0.92)**		
Reader 7	0.67 (0.50–0.79)	**0.93 (0.88–0.96)**	**0.89 (0.82–0.93)**	**0.88 (0.81–0.93)**	**0.91 (0.85–0.94)**	**0.84 (0.74–0.90)**	
Reader 8	0.30 (0.06–0.51)	0.43 (0.20–0.61)	0.49 (0.28–0.66)	0.57 (0.37–0.72)	0.47 (0.25–0.64)	0.42 (0.19–0.60)	0.49 (0.28–0.66)
**B. MOAKS.**							
Reader 2	0.65 (0.47–0.77)						
Reader 3	0.65 (0.47–0.77)	**0.74 (0.60–0.84)**					
Reader 4	**0.71 (0.56–0.81)**	**0.73 (0.59–0.83)**	**0.75 (0.62–0.84)**				
Reader 5	0.61 (0.42–0.74)	**0.81(0.71–0.88)**	**0.72 (0.57–0.82)**	**0.79 (0.67–0.87)**			
Reader 6	0.59 (0.40–0.73)	0.62 (0.43–0.75)	0.68 (0.51–0.79)	**0.72 (0.57–0.82)**	0.54 (0.33–0.70)		
Reader 7	0.64 (0.46–0.77)	**0.77 (0.64–0.85)**	0.62(0.44–0.76)	**0.72 (0.57–0.82)**	0.67 (0.50–0.79)	0.64 (0.47–0.77)	
Reader 8	0.33 (0.08–0.54)	0.59 (0.40–0.73)	0.55 (0.35–0.70)	0.48 (0.26–0.65)	0.64 (0.46–0.77)	0.42 (0.19–0.61)	0.31 (0.07–0.53)

Acceptable reliability for Synovitis-Effusion baseline status score (ICC > 0.80) using KIMRISS was achieved for 8 of 14 pair-wise comparisons of experienced readers and for 5 of 13 pair-wise comparisons that included at least one inexperienced reader (Table 4). For MOAKS, this target was attained in only two pair-wise comparisons (Table 4). Spearman’s Rank correlations between KIMRISS and MOAKS status scores varied from 0.42 to 0.82 among all the reader pairs and all were highly significant (*p* < 0.0001) (supplementary Table 3). Acceptable reliability for change from baseline to 1-year score of Synovitis-Effusion (ICC > 0.70) was achieved for all 14 pair-wise comparisons of experienced readers using KIMRISS and, also, for 12 of the 13 pair-wise comparisons that included an inexperienced reader (Table 5). For MOAKS Synovitis-Effusion change score, acceptable reliability was achieved for 1 of 14 pair-wise comparisons between experienced readers and for none of the 13 pair-wise comparisons that included an inexperienced reader (Table 5). Spearman’s Rank correlations between KIMRISS and MOAKS change scores varied from 0.24 to 0.67 among all the reader pairs and most were highly significant (*p* < 0.0001) (supplementary Table 7).

**Table 4.** Inter-reader reliability for status (baseline scan) score in Synovitis-Effusion using the KIMRISS (A) and MOAKS (B) MRI scoring platforms in 60 cases selected from the Osteoarthritis Initiative database. Values in the table reflect pair-wise intraclass correlation coefficients (95% confidence intervals) and bolded values are those which attain the pre-specified target for acceptable reliability of > 0.80. Readers 1 and 8 are inexperienced readers.

	Reader 1	Reader 2	Reader 3	Reader 4	Reader 5	Reader 6	Reader 7
**A. KIMRISS.**							
Reader 2	**0.93 (0.88–0.96)**						
Reader 3	**0.90 (0.83–0.94)**	**0.86 (0.73–0.93)**					
Reader 4	**0.89 (0.43–0.96)**	**0.84 (0.21–0.95)**	**0.93 (0.84–0.97)**				
Reader 5	**0.90 (0.77–0.95)**	**0.93 (0.87–0.96)**	**0.82 (0.54–0.92)**	0.78 (0.04–0.93)			
Reader 6	0.60 (–0.07–0.85)	0.56 (–0.09–0.84)	0.67 (0.06–0.86)	0.76 (0.29–0.90)	0.51 (–0.09–0.81)		
Reader 7	**0.87 (0.52–0.95)**	**0.90 (0.71–0.95)**	**0.81 (0.26–0.93)**	0.76 (–0.06–0.93)	**0.91 (0.85–0.95)**	0.48 (–0.09–0.80)	
Reader 8	0.66 (–0.05–0.87)	0.67 (0.00–0.88)	0.60 (–0.1–0.85)	0.54 (–0.1–0.84)	0.73 (0.20–0.89)	0.31 (–0.08–0.67)	0.79 (0.47–0.90)
**B. MOAKS.**							
Reader 2	0.59 (0.37–0.74)						
Reader 3	**0.81 (0.70–0.88)**	0.67 (0.48–0.79)					
Reader 4	0.75 (0.62–0.85)	0.66 (0.41–0.80)	0.78 (0.66–0.86)				
Reader 5	0.72(0.57–0.82)	0.68 (0.51–0.79)	0.74 (0.60–0.84)	**0.80 (0.68–0.87)**			
Reader 6	0.48 (0.24–0.66)	0.47 (0.00–0.73)	0.49 (0.23–0.67)	0.58 (0.37–0.73)	0.51(0.23–0.69)		
Reader 7	0.59 (0.26–0.77)	0.71 (0.55–0.81)	0.67(0.37–0.82)	0.67 (0.23–0.84)	0.75 (0.53–0.86)	0.48 (–0.08–0.76)	
Reader 8	0.48 (0.26–0.66)	0.53 (0.32–0.69)	0.64 (0.46–0.77)	0.53 (0.32–0.69)	0.62 (0.44–0.75)	0.38 (0.15–0.58)	0.51 (0.29–0.68)

**Table 5.** Inter-reader reliability for baseline to 1-year change in Synovitis-Effusion score using the KIMRISS (A) and MOAKS (B) MRI scoring platforms in 60 cases selected from the Osteoarthritis Initiative database. Values in the table reflect pair-wise intraclass correlation coefficients (95% confidence intervals) and bolded values are those which attain the pre-specified target for acceptable reliability of > 0.70. Readers 1 and 8 are inexperienced readers.

	Reader 1	Reader 2	Reader 3	Reader 4	Reader 5	Reader 6	Reader 7
**A. KIMRISS.**							
Reader 2	**0.86 (0.78–0.92)**						
Reader 3	**0.83 (0.73–0.90)**	**0.88 (0.81–0.93)**					
Reader 4	**0.89 (0.82–0.93)**	**0.89 (0.82–0.93)**	**0.91 (0.85–0.94)**				
Reader 5	**0.89 (0.82–0.93)**	**0.95 (0.91–0.97)**	**0.88 (0.80–0.92)**	**0.92(0.87–0.95)**			
Reader 6	**0.80 (0.68–0.87)**	**0.84 (0.75–0.90)**	**0.83 (0.73–0.90)**	**0.86 (0.78–0.91)**	**0.85 (0.76–0.91)**		
Reader 7	**0.90 (0.84–0.94)**	**0.88 (0.81–0.93)**	**0.85 (0.77–0.91)**	**0.87 (0.79–0.92)**	**0.88 (0.81–0.93)**	**0.81 (0.70–0.88)**	
Reader 8	**0.77 (0.65–0.86)**	**0.78 (0.65–0.86)**	**0.76 (0.63–0.85)**	**0.78 (0.66–0.87)**	**0.81 (0.70–0.88)**	0.69 (0.53–0.80)	**0.79 (0.68–0.87)**
**B. MOAKS.**							
Reader 2	0.40 (0.16–0.59)						
Reader 3	0.50 (0.22–0.67)	0.54 (0.33–0.70)					
Reader 4	0.43 (0.20–0.62)	0.44 (0.21–0.62)	0.59 (0.40–0.73)				
Reader 5	0.42 (0.12–0.61)	0.58 (0.38–0.72)	0.59 (0.40–0.74)	0.55 (0.35–0.71)			
Reader 6	0.39 (0.15–0.58)	0.28 (0.03–0.50)	0.43 (0.20–0.62)	0.20 (–0.06–0.43)	0.36 (0.12–0.56)		
Reader 7	0.55 (0.35–0.71)	0.48 (0.25–0.65)	0.67 (0.50–0.79)	0.42 (0.19–0.61)	0.68 (0.52–0.80)	0.55 (0.35–0.71)	
Reader 8	0.40 (0.16–0.59)	0.53 (0.32–0.69)	0.64 (0.46–0.77)	0.65 (0.47–0.77)	**0.73 (0.58–0.83)**	0.28 (0.03–0.50)	0.64 (0.47–0.77)

**Figure 7.**
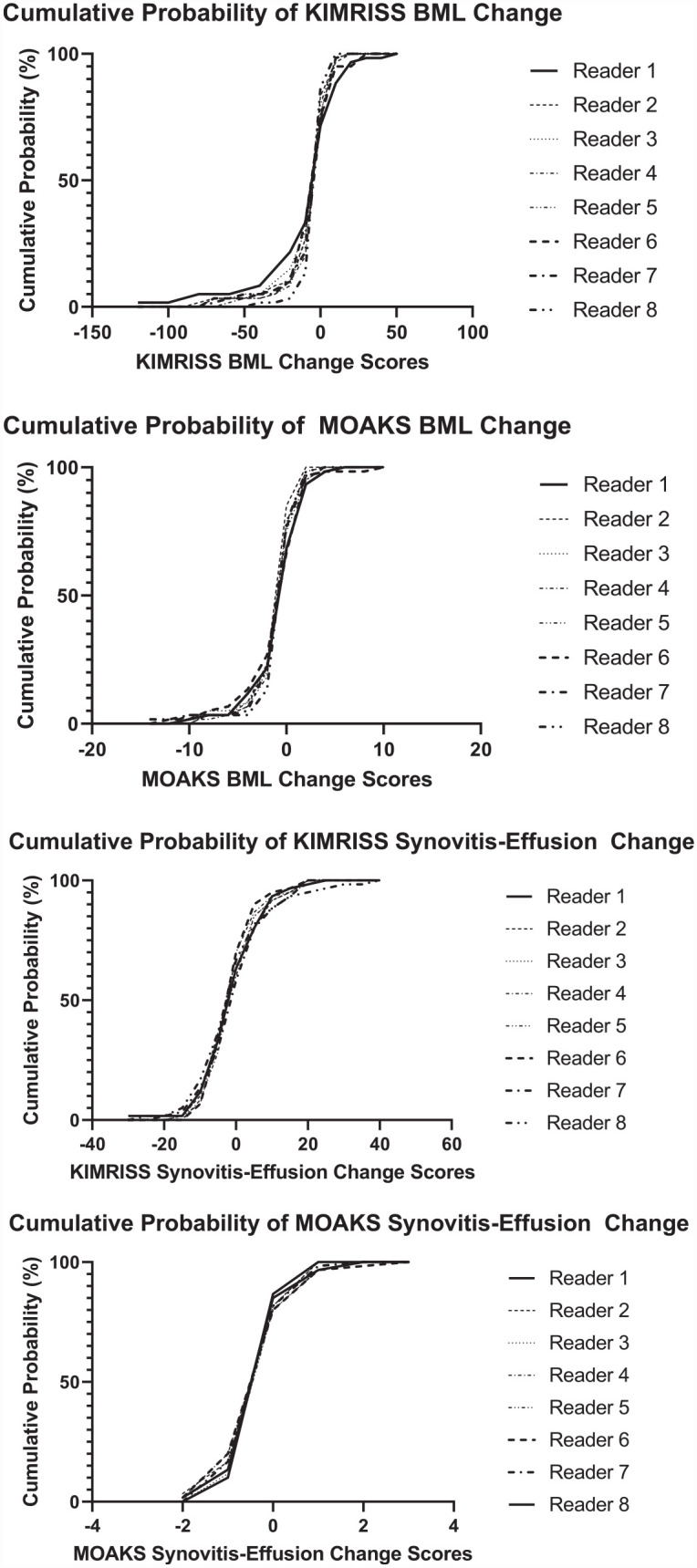
Cumulative probability plots for all 8 readers for baseline to 1-year change in KIMRISS BML, MOAKS BML, KIMRISS synovitis-effusion, and MOAKS synovitis-effusion scores from MRI scans of 60 cases from the Osteoarthritis Initiative.

Figure 7 illustrates the reliability of scores across the whole range of change scores according to individual reader data using cumulative probability plots. The plots for all readers were reasonably superimposable for both methods though not directly comparable due to differences in scoring ranges. These plots also demonstrate that while the mean change in BML and synovitis score over 1 year was minimal, more substantial change for BML and/or S-E was evident in 20%–30% of patients, and this was more discernable on the KIMRISS plots.

Reliability across all 6 experienced readers for baseline status and baseline to 1 year change scores was superior using the KIMRISS versus the MOAKS methods for both BML and Synovitis-Effusion (Table 6).

**Table 6.** Summary of reliability for semi-quantitative assessment of MRI bone marrow lesions and synovitis-effusion according to the KIMRISS and MOAKS scoring methods by expert readers evaluating MRI scans from 60 cases obtained from the Osteoarthritis Initiative.

MRI feature	Method/scoring assessment	Expert reader ICC (95% CI)	Expert reader pairs Mean (SD)	Expert reader pairs median (range)
BML	KIMRISS/status	0.91 (0.88-0.94)	0.92 (0.04)	0.93 (0.81–0.97)
	KIMRISS/change	0.88 (0.83–0.92)	0.87 (0.04)	0.88 (0.80–0.93)
	MOAKS/status	0.67 (0.56–0.77)	0.68 (0.09)	0.70 (0.50–0.82)
	MOAKS/change	0.69 (0.60–0.78)	0.70 (0.07)	0.72 (0.54–0.81)
	MOAKS/WG status*	0.70 (0.60–0.79)	0.71 (0.07)	0.72 (0.57–0.82)
	MOAKS/WG change*	0.70(0.60–0.79)	0.70 (0.04)	0.69 (0.64–0.77)
Synovitis–Effusion	KIMRISS/status	0.75 (0.52–0.86)	0.77 (0.15)	0.80 (0.43–0.93)
	KIMRISS/change	0.87 (0.82–0.91)	0.87 (0.04)	0.88 (0.81–0.95)
	MOAKS status	0.65 (0.52–0.75)	0.64 (0.11)	0.67 (0.47–.80)
	MOAKS change	0.48 (0.37–0.60)	0.49 (0.14)	0.52 (0.20–0.68)

* Within grade scoring based on 5 expert reader data.

### Construct validation of KIMRISS and MOAK MRI scores for BML and synovitis-effusion

The mean (SD) change in WOMAC pain over 1 year was -1.0 (3.7). Significant, though weak, correlations were observed between baseline scores for BML and baseline WOMAC pain score, there being little difference between the KIMRISS and MOAKS methods (Table 7). Similar correlations were observed between baseline to 1 year change in WOMAC pain and change in KIMRISS or MOAKS BML scores, there being little difference between the methods. We tested whether change in KIMRISS or MOAKS BML score were independently associated with 1-year change in WOMAC pain using multivariate linear regression adjusted for age, gender, BMI, and Kelgren-Lawrence grade but no association was observed.

**Table 7.** Correlations between KIMRISS and MOAKS MRI scores for BML and Synovitis-Effusion with WOMAC pain scores.

Method*	WOMAC Pain status	WOMAC Pain change
KIMRISS BML status	**0.33** *p* = 0.016	–0.13 *p* = 0.35
KIMRISS BML change	–0.10 *p* = 0.46	**0.31** *p* = 0.026
MOAKS BML status	**0.27** *p* = 0.048	–0.11 *p* = 0.41
MOAKS BML change	–0.017 *p* = 0.90	**0.30** *p* = 0.027
MOAKS BML WG status	**0.29** *p* = 0.035	–0.09 *p* = 0.52
MOAKS BML WG change	0.029 *p* = 0.84	**0.20** *p* = 0.15
KIMRISS Synovitis-Effusion status	0.37 *p* = 0.0064	0.12 *p* = 0.38
KIMRISS Synovitis-Effusion change	–0.11 *p* = 0.45	0.22 *p* = 0.12
MOAKS Synovitis-Effusion status	0.43 *p* = 0.0012	0.10 *p* = 0.47
MOAKS Synovitis-Effusion change	–0.14 *p* = 0.30	0.27 *p* = 0.051

* MRI reads of 6 experienced readers.

Somewhat stronger correlations were observed between baseline WOMAC Pain score and baseline scores for MRI Synovitis-Effusion, with little difference between the methods (Table 7). Correlations with change in WOMAC pain over 1 year were not significant for either method.

### Feasibility of KIMRISS and MOAKS scores

**Figure 8.**
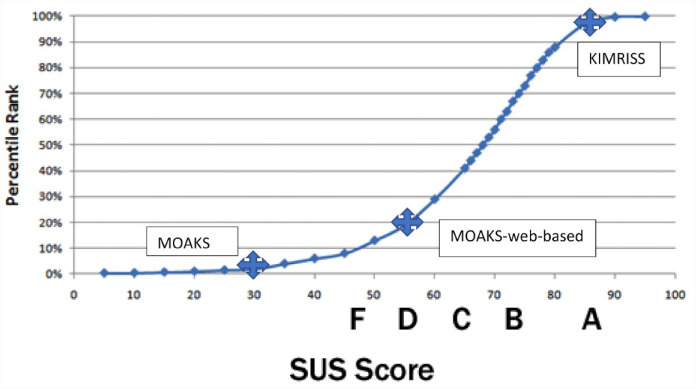
Percentile rankings of SUS scores based on more than 5000 SUS observations^37^ and mean SUS scores and percentile rankings for 8 readers using the KIMRISS and MOAKS methods to score MRI scans of 60 cases from the Osteoarthritis Initiative.

Mean reading time per case was 13.5 min for KIMRISS and 10.4 min for MOAKS. SUS scores were available for 6 experienced and 1 inexperienced reader. Consistently high SUS scores were noted for the KIMRISS method (Table 8), irrespective of prior reader experience with the scoring methods. SUS scores from all readers were at least greater than the 80th centile for ranking of usability and the mean usability score of 85.7 was at the 95th centile of ranking for usability (Figure 8). SUS scores were considerably and consistently lower for the MOAKS method, although the web-based MOAKS method had consistently higher scores than the conventional MOAKS method. The mean SUS score for MOAKS was at only the 20th centile ranking for usability.

**Table 8.** System Usability Scores for the KIMRISS, MOAKS, and web-based MOAKS scoring methods for semiquantitative assessment of BML and Synovitis-Effusion on MRI scans from the Osteoarthritis Initiative.

Readers	KIMRISS	MOAKS	MOAKS (web-based)
1	85	10	52.5
2	87.5	42.5	55
3	87.5	37.5	72.5
4	82.5	20	47.5
5	87.5	37.5	55
6	92.5	30	52.5
7	77.5	30	52.5
Mean	**85.7**	**29.6**	**55.4**

## Discussion

The developers of the KIMRISS method have created a revised version of this scoring platform and conducted a comparative validation analysis with the method that is currently considered to be the most widely used to assess BML and S-E, the MOAKS method. The KIMRISS method scored consistently highly for feasibility, irrespective of reader expertise, when assessed by the System Usability Scale. By comparison, the MOAKS method was considered non-feasible without the added availability of tools that permit ease of delineation of the boundaries of subregions as well as web-based direct data entry. The KIMRISS method also performed favorably compared to the MOAKS method for reliability, whether assessed by the ICC or the SDC (as a percentage of the maximum score). Pre-specified targets for acceptable reader reliability were achieved for both KIMRISS BML and S-E for 1-year change scores for all expert reader pairs and even for some reader pairs that included inexperienced readers. This was especially noteworthy as the amount of change over 1 year was very small. Construct validity versus WOMAC pain was comparable for both scoring methods. Performance for sensitivity to change could not be determined because of the small degree of change in BML and S-E over the 1-year time frame between scans.

We designed the KIMRISS scoring system to focus on MRI biomarkers for OA that could reflect potentially reversible processes such as inflammation and/or vascularization, especially in the shorter time frame of placebo-controlled trials. Moreover, KIMRISS evaluates BML and S-E, which relate more strongly to symptoms than other MRI features such as cartilage changes.^1,2,41^ Histopathological analyses of BML are limited to samples obtained from patients undergoing arthroplasty for severe OA. A recent systematic review compared histological and morphometrical changes underlying subchondral bone abnormalities in inflammatory and degenerative musculoskeletal disorders.^42^ Thirteen studies (309 patients) were identified up to September 2017 correlating BMLs in OA with histopathological changes, of which eight studied knee OA,^43–50^ and five studied hip OA.^51–54^ Abnormalities included increased bone remodeling, thickening of the subchondral plate, increase in trabecular number, volume, and thickness, focal areas of swelling and disintegration of fat cells, areas of cell apoptosis or necrosis, inflammatory cell infiltration, and partial replacement of adipose-type marrow with fibrous or fibrovascular tissue.

It has recently been demonstrated that BML may be associated with increased vascularity suggesting a reparatory response to microtrauma. One study investigated the relationship between BMLs in the tibial plateau (TP) of knee OA and bone matrix microdamage, osteocyte density and vascular changes in 73 patients undergoing knee arthroplasty.^55^ When compared to NO-BML tissue obtained from anatomically matched sites, marrow tissue within BML zones had greater density and length of vascular channels and there was an increased density of microdamage in both the subchondral plate and the trabeculae. A four-fold increase in angiogenesis markers has been reported in BMLs in hip OA^53^ and a gene expression study reported increased vascular proliferation within BML zones, accompanied by genes in the angiogenic pathway being among the most upregulated genes in BMLs.^50^

This ‘repair hypothesis’ is supported by studies showing that the natural course of subchondral bone abnormalities is very variable with fluctuation in size, and even regression.^56–59^ We have previously reported that BML may change within an 8-week window in patients with hip OA undergoing imaging-guided intra-articular injections of steroid.^60^ This repair hypothesis is further supported by limited data from therapeutic studies of patients with knee OA and BML where drugs targeting bone remodeling, such as bisphosphonates and strontium ranelate, led to a reduction in size of BMLs, reduced pain and cartilage loss, and delayed need for total knee replacement.^13,14^ In addition, tumor necrosis factor inhibitors, which may reduce vascularization, have also been shown to reduce BMLs in OA.^61^

It is also possible that angiogenesis contributes to structural damage. There is in vitro evidence for increased expression of vascular endothelial growth factor (VEGF) in chondrocytes by biomechanical stimulation and a direct role for increased VEGF expression in cartilage degeneration.^62,63^ Inhibition of synovial angiogenesis has also been suggested as a novel treatment approach to control inflammation and pain in OA by reducing damage to subchondral bone and cartilage.^64,65^ The accumulating data therefore supports the notion that OA is associated with modifiable factors and an early focus on reducing joint loading, the use of therapies that reduce joint remodeling and angiogenesis, and using BML as an outcome measure, might provide effective intervention for the development of OA.

We have reported a preliminary study comparing the performance of the first version of the KIMRISS method versus the current standard methodology, the MOAKS. In the first report, where 2 experienced and 2 inexperienced readers assessed baseline and 1 year MRI scans from 80 cases of the OAI cohort, reliability for status and change scores was greater for the KIMRISS method as determined by using either the ICC or the SDC metric.^24^ However, readers reported concerns with the feasibility of both methods. For KIMRISS, positioning of the overlays lacked precision for the different contours of the articulating bones and required frequent repositioning. For version 2 of KIMRISS, the overlays were redesigned to fit the contours of articulating bone more accurately. Furthermore, the implementation of anchor overlays at the most lateral and medial edges of the femoral condyles, the tibial plateau, and the patella meant that repositioning of overlays was no longer necessary. The success of this endeavor is highlighted by the high scores on the SUS scale for feasibility and the even more favorable data for reliability observed with the ICC and SDC metrics in this scoring exercise. But it was also felt necessary to develop a scoring overlay to enhance the feasibility of scoring BML with the MOAKS method and thereby conduct a more appropriate comparison of the two scoring methods. As expected, both reliability and feasibility improved when the MOAKS overlays were used in this scoring exercise. Nevertheless, we consistently demonstrate that a scoring method based on many simple binary scoring decisions (BML yes/no), as in KIMRISS, performs more reliably than the fewer but more challenging scoring decisions in MOAKS, which requires readers to estimate the percentage volume involved by BML and cystic change within more anatomically complex regions, each comprised of a three-dimensional construct.

There are several study limitations. First is the bias inherent to a comparative analysis designed by the developers of a new method. In particular, comparisons between the reliability of the KIMRISS and MOAKS methods are limited by the lack of availability of a MOAKS RETIC module with MOAKS developer scores embedded in the scoring interface that would have allowed the calibration process to be the same between the two scoring methods. Sufficient knowledge transfer tools can be helpful in ensuring appropriate and consistent use of an imaging-based scoring method. Nevertheless, we attempted to account for this bias toward the KIMRISS method by developing both an electronic overlay as well as web-based data entry for the MOAKS method to enhance feasibility and scoring performance, neither of which have been available to date. Sensitivity to change could not be readily assessed because the degree of change in BML and S-E was very small over the one-year time frame of follow up selected for this exercise. However, KIMRISS was more responsive than MOAKS in demonstrating change in BML in a small group of patients with knee OA and clinical evidence of knee effusion who received a 12-week course of a TNFi, adalimumab.^24^ Setting the anchors for scoring BML with KIMRISS is time consuming. Feasibility could be improved by automating linkage between the overlays and the articulating joint margins. It is possible that BML concentrated in one bone or region, especially subchondral bone, may be more clinically meaningful than BML spread through a joint. In particular, the value of assessing non-articular bone may be questioned in the setting of OA. However, data analytics in much larger longitudinal and clinical trial datasets can address which subregions assessed in KIMRISS may be most sensitive to change. Furthermore, analysis of both subchondral and non-articular bone increases the potential applicability of the KIMRISS system to other disease processes such as inflammatory arthropathies and avascular necrosis, in which non-subchondral BML may be highly clinically meaningful.

In conclusion, we have designed a web-based method for semi-quantitative scoring of BML and S-E, KIMRISS, which demonstrates a high degree of feasibility, as assessed by the System Usability Scale, and reliability when compared to the current method used for OA, the MOAKS method. Pre-specified targets for acceptable reader reliability were achieved for both KIMRISS BML and S-E for 1-year change in BML scores for all expert reader pairs and even for some reader pairs that included inexperienced readers. This was especially noteworthy as the amount of change in BML over 1 year was very small. Construct validity versus WOMAC pain was comparable for both scoring methods. We also created an enhanced web-based scoring interface for MOAKS that simplifies delineation of the boundaries of subregions as well as permits direct web-based data entry. Further assessment for sensitivity to change will require the availability of therapeutic agents that demonstrate efficacy in OA.

## Supplemental Material

sj-docx-1-tab-10.1177_1759720X231171766 – Supplemental material for Comparative validation of the knee inflammation MRI scoring system and the MRI osteoarthritis knee score for semi-quantitative assessment of bone marrow lesions and synovitis-effusion in osteoarthritis: an international multi-reader exercise Click here for additional data file. Supplemental material, sj-docx-1-tab-10.1177_1759720X231171766 for Comparative validation of the knee inflammation MRI scoring system and the MRI osteoarthritis knee score for semi-quantitative assessment of bone marrow lesions and synovitis-effusion in osteoarthritis: an international multi-reader exercise by Walter P. Maksymowych, Jacob L. Jaremko, Susanne J. Pedersen, Iris Eshed, Ulrich Weber, Andrew McReynolds, Paul Bird, Stephanie Wichuk and Robert G. Lambert in Therapeutic Advances in Musculoskeletal Disease

## Declarations

**Ethics approval and Consent to participate:** Not applicable

**Consent for publication:** Not applicable.

**Availability of data and materials:** The OAI database is publicly accessible. Free access to the KIMRISS and MOAKS reading platforms is available for academic users by registering at www.carearthritis.com
