# Supplementary material for: Comparative validation of the knee inflammation MRI scoring system and the MRI osteoarthritis knee score for semi-quantitative assessment of bone marrow lesions and synovitis-effusion in osteoarthritis: an international multi-reader exercise
Source: Ther Adv Musculoskelet Dis. 2023 Jul 12;15:1759720X231171766. doi: 10.1177/1759720X231171766 (PMC10345937; doi:10.1177/1759720X231171766)
Supplement: sj-docx-1-tab-10.1177_1759720X231171766 – Supplemental material for Comparative validation of the knee inflammation MRI scoring system and the MRI osteoarthritis knee score for semi-quantitative assessment of bone marrow lesions and synovitis-effusion in osteoarthritis: an international multi-reader [file sj-docx-1-tab-10.1177_1759720X231171766.docx]

**SUPPLEMENTARY DATA**

**MOAKS Scoring Methodology**

MOAKS BML is scored in 15 regions (2 patellar, 6 femoral, 7 tibial), based on BML size (none=0, <33% of region=1; 33–66% of region=2, >66%=3), for a maximum score 15 x 3=45 per knee^22^. The patella is divided into two sub-regions, the medial and lateral patella on the axial view. The femur is divided into 6 sub-regions, medial and lateral trochlea, medial and lateral central femur, and the medial and lateral posterior femur. The tibia is divided into three medial (anterior, central and posterior) and three lateral (anterior, central and posterior) subregions covered by articular cartilage, and the subspinous subregion [SS-region delineated by the tibial spines]. MOAKS also scores the percentage of BML that is non-cystic and the number of BML in a region although each region generates a single grade for size inclusive of all BMLs into one score. Synovitis-effusion and Hoffa’s synovitis is assessed qualitatively according to a 0-3 grade on axial images that is based on the degree of distension of the medial and lateral patellar recesses: Grade 0: Normal intra-articular hyperintensity; Grade 1: Fluid continuous in the retropatellar space; Grade 2: Slight convexity of the suprapatellar bursa; Grade 3: Evidence of capsular distention. The methodology has been described in detail in previous publications^22^.

**TABLES**

**Table 1. Baseline patient characteristics of 60 cases from the OAI dataset with baseline and 1-year follow up MRI scans.**

| **Age (mean(SD))** | **61.9 (8.8)** |
| --- | --- |
| **Males (%)** | **16 (26.7%)** |
| **Symptom duration (mean(SD))** | **not available** |
| **WOMAC pain (0-20)*** | **12.3 (11.9)** |
| **WOMAC function. (0-68)**** | **3.9 (4.3)** |
| **Kellgren-Lawrence grade** | **1.4 (0.7)** |
| **0 n (%)** | **6 (10%)** |
| **1 n (%)** | **22 (36.7%)** |
| **2 n(%)** | **32 (53.3%)** |
| **3 n(%)** | **n/a** |
| **4 n(%)** | **n/a** |

*** n=53**

**** n=54**

**Table 2.** Inter-reader reliability for status (baseline scan) score in BML using the MOAKS MRI scoring method that includes within grade scoring of change in BML, in 60 cases selected from the Osteoarthritis Initiative database. Values in the table reflect pair-wise intraclass correlation coefficients (95% confidence intervals) and bolded values are those which attain the pre-specified target for acceptable reliability of >0.80. Readers 1 and 8 are inexperienced readers.

|  | **Reader 1** | **Reader 2** | **Reader 3** | **Reader 4** | **Reader 5** | **Reader 6** | **Reader 7** |
| --- | --- | --- | --- | --- | --- | --- | --- |
| **Reader 2** | 0.54 (0.18-0.74) |  |  |  |  |  |  |
| **Reader 3** | 0.61 (0.27-0.78) | 0.59 (0.40-0.74) |  |  |  |  |  |
| **Reader 4** | 0.73 (0.57-0.84) | 0.72 (0.46-0.85) | 0.72 (0.53-0.83) |  |  |  |  |
| **Reader 5** | 0.55 (0.15-0.76) | 0.76 (0.63-0.85) | 0.72 (0.57-0.82) | 0.74 (0.41-0.87) |  |  |  |
| **Reader 6*** |  |  |  |  |  |  |  |
| **Reader 7** | 0.51 (0.10-0.73) | **0.82 (0.72-0.89)** | 0.57 (0.37-0.72) | 0.71 (0.29-0.86) | 0.72 (0.58-0.82) |  |  |
| **Reader 8** | 0.28 (-0.05-0.54) | 0.55 (0.19-0.75) | 0.59 (0.40-0.74) | 0.72 (0.46-0.85) | 0.76 (0.63-0.85) |  | 0.55 (031-0.72) |

*****Reader 6 did not conduct within grade scoring of BML.

**Table 3.** Correlations between KIMRISS and MOAKS for baseline BML score (Spearman’s Rank)

|  | **KIMRISS BML BL** | | | | | | | |
| --- | --- | --- | --- | --- | --- | --- | --- | --- |
| **MOAKS BML BL** | Reader 1 | Reader 2 | Reader 3 | Reader 4 | Reader 5 | Reader 6 | Reader 7 | Reader 8 |
| Reader 1 | 0.75  P<0.0001 | 0.72  P<0.0001 | 0.76  P<0.0001 | 0.775  P<0.0001 | 0.71  P<0.0001 | 0.71  P<0.0001 | 0.75  P<0.0001 | 0.60  P<0.0001 |
| Reader 2 | 0.66  P<0.0001 | 0.70  P<0.0001 | 0.68  P<0.0001 | 0.70  P<0.0001 | 0.66  P<0.0001 | 0.67  P<0.0001 | 0.69  P<0.0001 | 0.49  P=0.0001 |
| Reader 3 | 0.75  P<0.0001 | 0.71  P<0.0001 | 0.73  P<0.0001 | 0.74  P<0.0001 | 0.74  P<0.0001 | 0.70  P<0.0001 | 0.72  P<0.0001 | 0.57  P<0.0001 |
| Reader 4 | 0.68  P<0.0001 | 0.74  P<0.0001 | 0.70  P<0.0001 | 0.73  P<0.0001 | 0.70  P<0.0001 | 0.67  P<0.0001 | 0.71  P<0.0001 | 0.51  P<0.0001 |
| Reader 5 | 0.63  P<0.0001 | 0.68  P<0.0001 | 0.74  P<0.0001 | 0.72 P<0.0001 | 0.74  P<0.0001 | 0.62  P<0.0001 | 0.70 P<0.0001 | 0.53  P<0.0001 |
| Reader 6 | 0.56 P<0.0001 | 0.64  P<0.0001 | 0.58  P<0.0001 | 0.61  P<0.0001 | 0.55  P<0.0001 | 0.62  P<0.0001 | 0.61  P<0.0001 | 0.39  P=0.0019 |
| Reader 7 | 0.66  P<0.0001 | 0.72  P<0.0001 | 0.68  P<0.0001 | 0.72  P<0.0001 | 0.64  P<0.0001 | 0.74  P<0.0001 | 0.72  P<0.0001 | 0.44  P=0.0004 |
| Reader 8 | 0.55  P<0.0001 | 0.66  P<0.0001 | 0.74  P<0.0001 | 0.71  P<0.0001 | 0.69  P<0.0001 | 0.62  P<0.0001 | 0.66  P<0.0001 | 0.58  P<0.0001 |

**Table 4.** Inter-reader reliability for baseline to 1-year change in BML score using the MOAKS MRI scoring method that includes within grade scoring of change in BML, in 60 cases selected from the Osteoarthritis Initiative database. Values in the table reflect pair-wise intraclass correlation coefficients (95% confidence intervals) and bolded values are those which attain the pre-specified target for acceptable reliability of >0.80. Readers 1 and 8 are inexperienced readers.

|  | **Reader 1** | **Reader 2** | **Reader 3** | **Reader 4** | **Reader 5** | **Reader 6** | **Reader 7** |
| --- | --- | --- | --- | --- | --- | --- | --- |
| **Reader 2** | 0.63 (0.45-0.76) |  |  |  |  |  |  |
| **Reader 3** | 0.66 (0.49-0.78) | 0.69 (0.54-0.81) |  |  |  |  |  |
| **Reader 4** | 0.65 (0.48-0.77) | 0.68 (0.52-0.80) | **0.71 (0.55-0.81)** |  |  |  |  |
| **Reader 5** | 0.61 (0.42-0.75) | **0.77 (0.64-0.86)** | 0.68 (0.52-0.80) | 0.69 (0.53-0.80) |  |  |  |
| **Reader 6*** |  |  |  |  |  |  |  |
| **Reader 7** | 0.66 (0.49-0.78) | **0.77 (0.65-0.86)** | 0.64 (0.47-0.77) | **0.70 (0.54-0.81)** | 0.67 (0.50-0.79) |  |  |
| **Reader 8** | 0.32 (0.07-0.53 | 0.57 (0.37-0.72) | 0.51 (0.30-0.68) | 0.46 (0.24-0.64) | 0.64 (0.47-0.77) |  | 0.30 (0.05-0.51) |

*****Reader 6 did not conduct within grade scoring of BML.

**Table 5**. Correlations between KIMRISS and MOAKS for change in BML score from baseline to 1 year (Spearman’s Rank)

|  | **KIMRISS BML Change** | | | | | | | | |
| --- | --- | --- | --- | --- | --- | --- | --- | --- | --- |
| **MOAKS BML Change** | Reader 1 | Reader | Reader 3 | Reader 4 | Reader 5 | Reader 6 | Reader 7 | Reader 8 |  |
| Reader 1 | 0.55  P<0.0001 | 0.43  P=0.0007 | 0.46  P=0.0002 | 0.38  P=0.0027 | 0.35  P=0.0066 | 0.45  P=0.0004 | 0.44  P=0.0004 | 0.18  P=0.18 |  |
| Reader 2 | 0.51  P<0.0001 | 0.51  P<0.0001 | 0.48  P=0.0001 | 0.44  P=0.0004 | 0.45  P=0.0004 | 0.53  P<0.0001 | 0.50  P=0.0001 | 0.30  P=0.02 |  |
| Reader 3 | 0.50  P=0.0001 | 0.49  P=0.0001 | 0.43  P=0.0007 | 0.32  P=0.013 | 0.44  P=0.0005 | 0.37  P=0.0039 | 0.47  P=0.0002 | 0.42  P=0.001 |  |
| Reader 4 | 0.52  P<0.0001 | 0.59  P<0.0001 | 0.49  P=0.0001 | 0.52  P<0.0001 | 0.31  P=0.017 | 0.51  P<0.0001 | 0.47  P=0.0002 | 0.23  P=0.074 |  |
| Reader 5 | 0.46  P=0.0003 | 0.47  P=0.0002 | 0.45  P=0.0003 | 0.43  P=0.0005 | 0.49  P=0.0001 | 0.49  P=0.0001 | 0.40  P=0.0017 | 0.44  P=0.0004 |  |
| Reader 6 | 0.47  P=0.0001 | 0.44  P=0.0004 | 0.49  P=0.0001 | 0.39  P=0.0021 | 0.28  P=0.033 | 0.43  P=0.0007 | 0.39  P=0.0018 | 0.30  P=0.021 |  |
| Reader 7 | 0.38  P=0.0026 | 0.51  P<0.0001 | 0.37  P=0.0035 | 0.40  P=0.0016 | 0.31  P=0.015 | 0.40  P=0.0017 | 0.42  P=0.0009 | 0.28  P=0.032 |  |
| Reader 8 | 0.58  P<0.0001 | 0.54  P<0.0001 | 0.62  P<0.0001 | 0.58  P<0.0001 | 0.50  P=0.0001 | 0.42  P=0.0009 | 0.54  P<0.0001 | 0.48  P=0.0001 |  |

**Table 6.** Correlations between KIMRISS and MOAKS for baseline Synovitis-Effusion score (Spearman’s Rank)

|  | **KIMRISS Synovitis-Effusion BL** | | | | | | | |
| --- | --- | --- | --- | --- | --- | --- | --- | --- |
| **MOAKS Synovitis-Effusion BL** | **Reader 1** | **Reader 2** | **Reader 3** | **Reader 4** | **Reader 5** | **Reader 6** | **Reader 7** | **Reader 8** |
| **Reader 1** | 0.67  P<0.0001 | 0.63  P<0.0001 | 0.66  P<0.0001 | 0.72  P<0.0001 | 0.62  P<0.0001 | 0.70  P<0.0001 | 0.62  P<0.0001 | 0.71  P<0.0001 |
| **Reader 2** | 0.69  P<0.0001 | 0.68  P<0.0001 | 0.66  P<0.0001 | 0.73  P<0.0001 | 0.66  P<0.0001 | 0.73  P<0.0001 | 0.66  P<0.0001 | 0.71  P<0.0001 |
| **Reader 3** | 0.71  P<0.0001 | 0.69  P<0.0001 | 0.66  P<0.0001 | 0.73  P<0.0001 | 0.74  P<0.0001 | 0.74  P<0.0001 | 0.66  P<0.0001 | 0.73  P<0.0001 |
| **Reader 4** | 0.71  P<0.0001 | 0.71  P<0.0001 | 0.69  P<0.0001 | 0.76  P<0.0001 | 0.69  P<0.0001 | 0.70  P<0.0001 | 0.70  P<0.0001 | 0.75  P<0.0001 |
| **Reader 5** | 0.80  P<0.0001 | 0.76  P<0.0001 | 0.77  P<0.0001 | 0.82  P<0.0001 | 0.74  P<0.0001 | 0.82  P<0.0001 | 0.70  P<0.0001 | 0.76  P<0.0001 |
| **Reader 6** | 0.47  P=0.0001 | 0.49  P=0.0001 | 0.48  P=0.0001 | 0.54  P<0.0001 | 0.42  P=0.0009 | 0.54  P<0.0001 | 0.45  P=0.0003 | 0.56  P<0.0001 |
| **Reader 7** | 0.76  P<0.0001 | 0.77  P<0.0001 | 0.71  P<0.0001 | 0.77  P<0.0001 | 0.78  P<0.0001 | 0.77  P<0.0001 | 0.72  P<0.0001 | 0.77  P<0.0001 |
| **Reader 8** | 0.56  P<0.0001 | 0.53  P<0.0001 | 0.52  P<0.0001 | 0.59  P<0.0001 | 0.60  P<0.0001 | 0.58  P<0.0001 | 0.57  P<0.0001 | 0.57  P<0.0001 |

**Table 7.** Correlations between KIMRISS and MOAKS for baseline to 1-year change in Synovitis-Effusion score (Spearman’s Rank)

|  | **KIMRISS Synovitis-Effusion Change** | | | | | | | |
| --- | --- | --- | --- | --- | --- | --- | --- | --- |
| **MOAKS Synovitis-Effusion Change** | **Reader 1** | **Reader 2** | **Reader 3** | **Reader 4** | **Reader 5** | **Reader 6** | **Reader 7** | **Reader 8** |
| **Reader 1** | 0.51  P<0.0001 | 0.58  P<0.0001 | 0.52  P<0.0001 | 0.43  P=0.0006 | 0.54  P<0.0001 | 0.50  P=0.0001 | 0.54  P<0.0001 | 0.47  P=0.0001 |
| **Reader 2** | 0.47  P=0.0001 | 0.51  P<0.0001 | 0.50  P<0.0001 | 0.51  P<0.0001 | 0.52  P<0.0001 | 0.52  P<0.0001 | 0.45  P=0.0003 | 0.45  P=0.0003 |
| **Reader 3** | 0.61  P<0.0001 | 0.63  P<0.0001 | 0.60  P<0.0001 | 0.59  P<0.0001 | 0.63  P<0.0001 | 0.63  P<0.0001 | 0.55  P<0.0001 | 0.45  P=0.0003 |
| **Reader 4** | 0.46  P=0.0002 | 0.47  P=0.0001 | 0.39  P=0.0019 | 0.39  P=0.0019 | 0.47  P=0.0002 | 0.39  P=0.002 | 0.35  P=0.006 | 0.36  P=0.005 |
| **Reader 5** | 0.44  P=0.0005 | 0.50  P=0.0001 | 0.47  P=0.0002 | 0.45  P=0.0003 | 0.52  P<0.0001 | 0.42  P=0.0008 | 0.46  P=0.0002 | 0.44  P=0.0004 |
| **Reader 6** | 0.36  P=0.0051 | 0.37  P=0.0039 | 0.35  P=0.0063 | 0.34  P=0.0076 | 0.37  P=0.0041 | 0.24  P=0.068 | 0.37  P=0.0033 | 0.34  P=0.0072 |
| **Reader 7** | 0.65  P<0.0001 | 0.67  P<0.0001 | 0.65  P<0.0001 | 0.62  P<0.0001 | 0.67  P<0.0001 | 0.55  P<0.0001 | 0.54  P<0.0001 | 0.52  P<0.0001 |
| **Reader 8** | 0.47  P=0.0001 | 0.51  P<0.0001 | 0.45  P=0.0003 | 0.42  P=0.0008 | 0.53  P<0.0001 | 0.44  P=0.0004 | 0.46  P=0.0002 | 0.42  P=0.0009 |
